# Modulation of Oxidative Stress and Glycemic Control in Diabetic Wistar Rats: The Therapeutic Potential of Theobroma cacao and Camellia sinensis Diets

**DOI:** 10.7759/cureus.55985

**Published:** 2024-03-11

**Authors:** Edward Indla, KV Rajasekar, Bandarupalli Naveen Kumar, S. Saravana Kumar, Udaya Kumar P, Suresh Babu Sayana

**Affiliations:** 1 Department of Anatomy, Meenakshi Academy of Higher Education and Research, Chennai, IND; 2 Department of Radiology, Meenakshi Medical College Hospital and Research Institute, Chennai, IND; 3 Department of Anatomy, Mamata Medical College, Khammam, IND; 4 Department of Anatomy, Meenakshi Medical College Hospital and Research Institute, Chennai, IND; 5 Department of Pharmacology, Government Medical College and General Hospital, Suryapet, IND

**Keywords:** camellia sinensis, theobroma cacao, diabetic rats, glycemic control, oxidative stress

## Abstract

Background

Diabetes mellitus is a complex metabolic disorder characterized by oxidative stress and impaired glycemic control. This study investigates the therapeutic potential of *Theobroma cacao* and *Camellia sinensis* diets in diabetic Wistar rats and assesses their impact on oxidative stress markers and blood glucose levels.

Methods

In this experiment, eight groups of six male Wistar rats (n = 12.5%), aged 8 to 12 weeks, were carefully set up to see how different treatments for diabetes and oxidative stress affected the two conditions. The random selection process was implemented to minimize any potential bias and ensure that the results of the study would be representative of the general population of Wistar rats. The groups were as follows: a nondiabetic control group (NDC) served as the baseline, while diabetes was induced in the alloxan monohydrate group (150 mg/kg). Another group was given the standard drug metformin (M, 100 mg/kg), and two control groups that did not have diabetes were given extracts of *Theobroma cacao* (TC, 340 mg/kg) and *Camellia sinensis* (CS, 200 mg/kg). Three groups of diabetic rats were given a mix of these treatments. *Theobroma cacao* and *Camellia sinensis* extracts were given at set doses (TC, 340 mg/kg; CS, 200 mg/kg), along with 150 mg/kg of a drug that causes diabetes. Over a 21-day period, oxidative stress parameters such as glutathione (GSH), malondialdehyde (MDA), superoxide dismutase (SOD), and glutathione reductase (GSHrd) levels, and blood glucose were carefully measured to check for signs of oxidative stress and diabetes progression

Results

Considerable differences in GSH levels were noted across the groups, with the highest GSH concentration found in the group treated with the inducing drug, while the lowest GSH levels were observed in the diabetic group that was administered both *Theobroma cacao* and C*amellia sinensis* (p < 0.001). MDA levels also varied, with the diabetic group treated with *Theobroma cacao* having the highest MDA concentration (3.54 ± 0.29 μmol/L) and the nondiabetic control group treated with *Camellia sinensis *exhibiting the lowest MDA levels (1.66 ± 0.08 μmol/L; p < 0.001). SOD activity was highest in the standard drug group and lowest in the diabetic group treated with *Theobroma cacao*. GSH activity was notably higher in the diabetic groups that received dietary interventions (p < 0.001). Blood glucose levels showed diverse responses, with the standard drug group experiencing a substantial reduction, while the inducing drug group exhibited a consistent increase.

Conclusion

The study highlights the significant impact of dietary interventions with *Theobroma cacao *and *Camellia sinensis* on oxidative stress markers and blood glucose regulation in diabetic Wistar rats. These findings suggest a potential role for these dietary components in mitigating oxidative stress and improving glycemic control in diabetes, although further research is warranted to elucidate the underlying mechanisms and clinical implications.

## Introduction

Diabetes mellitus, a chronic metabolic disorder characterized by elevated blood glucose levels, has emerged as a substantial global health concern [1]. As reported by the International Diabetes Federation (IDF), the number of individuals living with diabetes exceeded 537 million worldwide in 2021, with projections indicating that this number may reach a staggering 643 million by 2030 if effective preventive measures and management strategies are not implemented [2]. Beyond the primary hallmark of disrupted glucose metabolism, diabetes is intricately intertwined with another critical factor: oxidative stress. Oxidative stress plays a pivotal role in driving the progression of diabetes-related complications [3].

Oxidative stress occurs when there is an imbalance between the production of reactive oxygen species (ROS) within the body and its ability to neutralize and detoxify these harmful molecules [4]. In the context of diabetes, this imbalance is particularly pronounced due to factors like hyperglycemia and other metabolic abnormalities. These factors lead to an upsurge in ROS production, disrupt the body's antioxidant defense mechanisms, and create a state of redox imbalance [5]. Consequently, this oxidative burden sets the stage for a cascade of events that exacerbate the pathophysiological processes associated with diabetes [6]. These processes include insulin resistance, dysfunction of pancreatic beta-cells responsible for insulin secretion, and the development of vascular complications [4, 5].

Effective management of diabetes necessitates not only the control of hyperglycemia but also the mitigation of oxidative stress [7]. While various pharmaceutical agents, such as metformin, have been employed to address glycemic control, their ability to combat oxidative stress remains somewhat limited. This limitation has prompted researchers to explore natural compounds and dietary interventions as potential adjuncts or alternatives to conventional diabetes management [7]. In another study, the authors investigated the neuroprotective effects of syringic acid (SA) in a rat model of Parkinson's disease (PD) induced by 6-hydroxydopamine (6-OHDA). SA is present in various foods such as Swiss chard, olives, walnuts, dates, spices, pumpkin, grapes, acai palm, honey, red wine, common pea, mung bean, rosemary, and dill. Known for its antioxidant and anti-inflammatory properties, SA was given orally to rats either before or after PD was induced through stereotaxic surgery. A number of tests were carried out to see how SA affected the amounts of substantia nigra (SN) dopamine, the ability of tyrosine hydroxylase (TH) and inducible nitric oxide synthase (iNOS), catatonia, and the immune responses to these substances. The study found that SA treatment significantly restored motor function, nigral dopamine release, and TH-positive cells in the SN while reducing iNOS expression and nitrite/nitrate levels. Additionally, SA increased the total antioxidant capacity and decreased the total oxidant capacity in the SN tissue [8]. These results suggest that SA has potential as a therapeutic agent for PD, offering neuroprotective, antioxidant, and anti-inflammatory benefits. Among these dietary constituents, *Theobroma cacao *(cocoa) and *Camellia sinensis* (green tea) have captured attention due to their antioxidant properties and the potential benefits they may offer in the context of diabetes [7].

*Theobroma cacao*, the cocoa tree, is native to the tropical regions of Mesoamerica and South America and is now cultivated globally in areas like West Africa, Southeast Asia, and Oceania, making it a vital economic crop. Belonging to the family Malvaceae, its seeds are processed into cocoa butter and chocolate. Historically, various parts of the plant, including seeds, pods, leaves, bark, and root, have been utilized. Seeds are central to chocolate production, while leaves and bark have been used in traditional medicine. Cocoa butter, derived from the beans, is prized in the cosmetics industry. *Theobroma cacao*'s cultivation and applications reflect its significance in both culinary and medicinal practices worldwide. *Theobroma cacao*, the plant source of cocoa and chocolate, boasts a rich content of bioactive compounds, including flavonoids and theobromine [8]. These compounds are well-known for their antioxidant properties and have been associated with improvements in insulin sensitivity and glucose metabolism [9]. Similarly, *Camellia sinensis*, the tea plant, is renowned for its high polyphenol content, with a particular emphasis on catechins. These catechins have demonstrated anti-inflammatory and antioxidant effects in various studies. Both *Theobroma cacao* [10] and *Camellia sinensis *[11] have shown promise in both animal and human research. They suggest potential in mitigating oxidative stress and enhancing glycemic control in individuals with diabetes.

This study aims to explore how diets containing *Theobroma cacao* (cocoa) and *Camellia sinensis* (tea) affect diabetic Wistar rats. It seeks to fill a gap in research by examining the specific effects of these plants on oxidative stress and blood sugar control in diabetic models. Although these plants are known for their antioxidant properties, there is still a lack of detailed studies comparing their effects on diabetes markers and understanding how they work biochemically. By carefully studying these dietary changes in diabetic rats, this research hopes to clarify how they impact oxidative stress and blood sugar levels, providing a better understanding of their potential benefits. Ultimately, the goal is to improve the use of natural dietary components in managing diabetes, addressing a critical research gap and contributing to advancements in treatment methods.

## Materials and methods

As an experiment, this study looked at how dietary foods that contain *Theobroma cacao* and *Camellia sinensis* can change oxidative stress and blood sugar levels in diabetic Wistar rats. It focused on the possible health benefits of these foods. The study was conducted at Vyas Labs in Hyderabad, Telangana, India. The study, conducted between February 10 and May 15, 2023, employed a randomized controlled trial (RCT) approach. This approach was chosen to enhance the internal validity of the study and minimize potential biases that could impact the results.

Preparation of *Camellia sinensis *extract

Indla et al. identified and authenticated the *Camellia sinensis* plant, and a voucher specimen (with the number GDC-32) was preserved for future reference [12]. The leaves of *Camellia sinensis* were meticulously harvested and sorted. These leaves were then air-dried under the shade for several weeks to reduce moisture content. Once dried, the leaves were ground into a fine powder using a mechanical grinder. The powdered leaves were then weighed and stored in an airtight container at a cool temperature (about 4°C) to preserve their freshness and potency until they were needed for further processing or analysis.

Preparation of *Theobroma cacao *seed extract

*Theobroma cacao* seeds were extracted, sorted, and air-dried under the shade for four weeks. The dried materials were ground using a mechanical grinder, weighed, and stored in an airtight container at 4°C until needed for further analysis.

Animal models and group categorization

For this study, male Wistar rats aged 8-12 weeks, weighing between 200 and 250 grams, were used. The animals were housed in a controlled environment with a 12-hour light/dark cycle, a temperature of 22 ± 2°C, and 50%-55% relative humidity. They had ad libitum access to food and water.

Sixty-four rats were randomly divided into eight groups, each consisting of eight animals: Group I, nondiabetic control, received a standard diet. Group II, diabetic model, administered with 150 mg/kg alloxan [12]. Group III was administered with 100 mg/kg metformin [12]. Group IV was treated with 340 mg/kg *Theobroma Cacao* extract [12]. Group V was treated with 200 mg/kg *Camellia Sinensis* extract [2, 12]. Group VI, diabetic model, was treated with 150 mg/kg alloxan and 340 mg/kg *Theobroma Cacao* extract. Group VII, diabetic model, was treated with 150 mg/kg alloxan and 200 mg/kg *Camellia Sinensis* extract. Group VIII, diabetic model, was treated with 150 mg/kg alloxan and combined treatments of *Theobroma Cacao* (340 mg/kg) and *Camellia Sinensis* (200 mg/kg) extracts (Table 1).

 

**Table 1:** Overview of the groups, their descriptions, the number of rats in each group, the type of treatment, and the corresponding dosage for each treatment

Group	Description	Number of rats	Treatment	Dosage
Group I	Nondiabetic control	8	Standard diet	None
Group II	Diabetic model	8	Alloxan administration	150 mg/kg of alloxan
Group III	Metformin treatment	8	Metformin	100 mg/kg of metformin
Group IV	*Theobroma cacao* extract treatment	8	*Theobroma cacao* extract	340 mg/kg of the extract
Group V	*Camellia sinensis* extract treatment	8	*Camellia sinensis *extract	200 mg/kg of the extract
Group VI	Diabetic model + *Theobroma cacao* extract	8	Alloxan +* Theobroma cacao* extract	150 mg/kg alloxan + 340 mg/kg *Theobroma cacao *extract
Group VII	Diabetic model + *Camellia sinensis* extract	8	Alloxan +* Camellia sinensis* extract	150 mg/kg alloxan + 200 mg/kg *Camellia sinensis* extract
Group VIII	Diabetic model + combined extract treatment	8	Alloxan + *Theobroma cacao* + *Camellia sinensis* extracts	150 mg/kg alloxan + 340 mg/kg *Theobroma cacao* + 200 mg/kg *Camellia sinensis* extracts

In this study, diabetes was induced using a freshly prepared solution of alloxan monohydrate. The solution was prepared by dissolving alloxan in normal saline, and rats that had fasted overnight (for 12 hours) were administered with the solution at a dose of 100 mg/kg body weight via intraperitoneal (IP) injection. We selected this method due to its rapid absorption and distribution properties, which are crucial for the effective induction of diabetes in our experimental setup. The IP route makes sure that alloxan monohydrate gets into the bloodstream more directly, which causes diabetes in the study population in a controlled and consistent way. Other routes, such as oral administration, could result in variable absorption rates due to metabolic processes in the digestive tract, potentially leading to inconsistent dosing and efficacy. To mitigate the effects of alloxan, the rats were treated with an orally administered 20% (w/v) glucose solution (10 mL) after six hours, followed by a 5% (w/v) glucose solution for 24 hours to prevent hypoglycemia. Rats that developed diabetes mellitus, as confirmed by glycosuria and hyperglycemia (blood glucose concentration exceeding 250 mg/dL), were selected for subsequent experimental tests [13].

The following are the eight treatment groups (n = 8) included in this study, to evaluate the clinical chemistry parameters according to the days of glucose estimation (day 0, 7, 14, 21): Group I, standard diet; Group II, alloxan administration 150 mg/kg/bw IP; Group III, metformin 100 mg/kg/bw PO; Group IV, *Theobroma cacao* extract 340 mg/kg/bw PO; Group V, *Camellia sinensis* extract 200 mg/kg/bw PO; Group VI, alloxan 100 mg/kg/bw + *Theobroma cacao* extract 340 mg/kg/bw PO; Group VII, alloxan 100 mg/kg/bw + *Camellia sinensis *extract 200 mg/kg/bw PO; and Group VIII, alloxan 100 mg/kg/bw + *Theobroma cacao *340 mg/kg/bw + *Camellia sinensis* extracts 200 mg/kg/bw PO (IP, intraperitoneal; bw, body weight; PO, orally).

The study included a comprehensive evaluation of clinical chemistry parameters to assess the impact of treatments on diabetic rats. Blood glucose levels were monitored at 0, 7, 14, and 21 days using a portable glucometer for accurate and timely measurements. To assess oxidative stress, key biochemical markers were analyzed: malondialdehyde (MDA) levels for lipid peroxidation, reduced glutathione (GSH), glutathione reductase (GSHrd) to gauge antioxidant defense, and superoxide dismutase (SOD) activity. These measurements were taken following the induction of diabetes with alloxan monohydrate and subsequent treatment with varying doses of *Theobroma cacao *and *Camellia sinensis* extracts over a 21-day period [14]. 

The 21-day period was selected for this study based on the time frame typically required to observe significant changes in the biochemical parameters being measured. This duration allows for the evaluation of both the acute and more prolonged effects of the treatments on diabetic rats.

In the context of diabetes research, a 21-day period is often chosen as it provides sufficient time for the development of diabetic symptoms following the induction of diabetes as well as for the potential therapeutic effects of treatments to manifest. It also matches the half-lives and metabolic rates of the biochemical markers being studied, like SOD, GSH, GSHrd, and MDA.

This amount of time makes sure that the data gathered is useful and shows how the treatments are affecting the body, which lets a full evaluation of how well they work to reduce complications related to diabetes be done.

Approval for the study was granted by the Institutional Animal Ethics Committee (IAEC/VL/16/2022-23). The research was carried out at Vyas Labs in Hyderabad, Telangana, India, during the period from February 10, 2023, to May 15, 2023.

Statistical analysis of the study involved the use of a one-way ANOVA, followed by Dunnett's T-test for further comparison. This approach allowed for group comparisons, with Dunnett's T-test used to contrast each experimental group's results with those of the control group. Significance was determined based on a p-value threshold of less than 0.05.

## Results

The study looked at how the diets of *Theobroma cacao* and *Camellia sinensis* affected oxidative stress markers and glucose control in Wistar rats that were diabetic. The findings are summarized in Table 2.

**Table 2:** Effect of Theobroma cacao and Camellia sinensis diets on oxidative stress markers and glycemic control in diabetic Wistar rats: summary of key findings Values are expressed as Mean ± SEM (n = 6), one-way ANOVA, followed by turkeys post hoc test. ***p < 0.001 compared to the diabetic control group. NDC: nondiabetic control; DC: diabetic control; TC:*Theobroma cacao;* CS: *Camellia sinensis*.

Experimental Group	GSH Levels (Mean ± SEM)	MDA Levels (Mean ± SEM)	SOD Activity (Mean ± SEM)	GSHrd Activity (Mean ± SEM)	Blood Glucose Levels (mg/dL): 0 Days (Mean ± SEM)	Blood Glucose Levels (mg/dL): 7 Days (Mean ± SEM)	Blood Glucose Levels (mg/dL): 14 Days (Mean ± SEM)	Blood Glucose Levels (mg/dL): 21 Days (Mean ± SEM)
Group I (NDC)	2.57 ± 0.03	2.04 ± 0.06	3.68 ± 0.04	7.66 ± 0.22	50 ± 5	50 ± 2	51 ± 7	57 ± 3
Group II (DC)	2.95 ± 0.09	2.32 ± 0.20	3.82 ± 0.12	7.79 ± 0.37	250 ± 5	260 ± 5	282 ± 10	343 ± 16
Group III (Metformin)	2.41 ± 0.25^***^	2.27 ± 0.12^***^	3.90 ± 0.11^***^	8.30 ± 0.67^***^	262 ± 12^***^	143 ± 8^***^	87 ± 9^***^	73 ± 9^***^
Group IV (NDC+ TC)	2.40 ± 0.06^***^	1.86 ± 0.02^***^	3.60 ± 0.08^***^	7.57 ± 0.12^***^	282±12^***^	269 ± 10^***^	208 ± 9^***^	178 ± 5^***^
Group V (NDC+ CS)	2.30 ± 0.03^***^	1.66 ± 0.08^***^	3.40 ± 0.06^***^	7.34±0.09^***^	279 ± 11^***^	262 ± 10^***^	214 ± 4^***^	183 ± 10^***^
Group VI (DC + TC)	1.01 ± 0.08^***^	3.54 ± 0.29^***^	2.07 ± 0.09^***^	13.19±0.14^***^	283 ± 12^***^	273 ± 9^***^	218 ± 3^***^	186 ± 6^***^
Group VII (DC + CS)	1.54 ± 0.16^***^	2.49 ± 0.13^***^	2.87 ± 0.18^***^	13.02±0.14^***^	295 ± 10^***^	281 ± 10^***^	236 ± 6^***^	195 ± 3^***^
Group VIII (DC+ TC + CS)	0.94 ± 0.10^***^	3.68 ± 0.18^***^	2.85 ± 0.07^***^	13.12±0.12^***^	303 ± 8^***^	292 ± 10^***^	238 ± 7^***^	195 ± 12^***^

GSH levels (µm GSH/mg protein) 

There were noticeable differences in the GSH levels across various groups. The group treated with the inducing drug exhibited the highest average GSH concentration, recorded at 2.95 ± 0.09 µm GSH/mg protein. Conversely, the group that received a combination of *Theobroma cacao* and *Camellia sinensis* (Group VIII) displayed the lowest level, with a mean concentration of 0.94 ± 0.10 µm GSH/mg protein, indicating a significant reduction (p < 0.001)

MDA levels (nmoles/dL)

The MDA levels were different in each group. Group VI, which was made up of diabetics who were treated with *Theobroma cacao*, had the highest level (3.54 ± 0.29 nmoles/dL). The lowest MDA levels were found in Group V, consisting of nondiabetic individuals who were administered with *Camellia sinensis*, with a concentration of 1.66 ± 0.08 nmoles/dL. This represented a significant decrease (p < 0.001).

SOD activity (units/100mg protein/min)

The standard drug group (Group III) had the most SOD activity, at 3.90 ± 0.11 units/100 mg protein/min. This was significantly higher (p < 0.001) than the other groups. The group of diabetics who were given *Theobroma cacao* had the lowest SOD activity (2.07 ± 0.09 units/100 mg protein/min; p < 0.001).

GSHrd activity (µmol of nicotinamide adenine dinucleotide phosphate (NADPH) oxidized/min/mg protein)

GSHrd activity exhibited a significant enhancement in the diabetic groups undergoing dietary interventions. Specifically, the diabetic group treated with *Theobroma cacao* (Group VI) demonstrated a substantial increase in GSHrd activity, reaching 13.19 ± 0.14 µmol of NADPH oxidized/min/mg protein, which was statistically significant (p < 0.001).

Blood glucose levels(mg/dL)

During the 21-day study period, different responses in blood glucose levels were noted. The group treated with the standard drug (Group III) saw a significant decrease in blood glucose levels, dropping from 262 ± 12 mg/dL initially to 73 ± 9 mg/dL by day 21 (p < 0.001). On the other hand, the group treated with the inducing drug (Group II) exhibited a steady rise in blood glucose levels, escalating to 343 ± 16 mg/dL by day 21 (p < 0.001).

## Discussion

The primary aim of this study was to evaluate the effects of *Theobroma cacao* and *Camellia sinensis* on oxidative stress markers and blood glucose regulation in diabetic Wistar rats. Our results underscore the potential of these natural substances as adjunctive treatments for diabetes mellitus, showcasing their ability to manage both oxidative stress and glycemic control. Alloxan, a common drug used to induce diabetes, creates a model that resembles type 1 diabetes by moderately destroying the β-cells in the islets of Langerhans, leading to reduced insulin release and subsequent hyperglycemia. The diabetogenic action of alloxan causes toxicity in pancreatic cells due to excess ROS, resulting in decreased insulin production, release, and secretion while also affecting organs such as the liver, kidney, and hematopoietic system. Decreased antioxidant enzyme levels and enhanced lipid peroxidation are well-documented in alloxan-induced diabetes [12-18].

Reduced GSH is a nonenzymatic antioxidant that scavenges free radicals produced within the biological system, protecting cells from oxidative damage. GSH is an intracellular thiol-rich tripeptide that helps maintain the proper balance of oxidative stress [15]. Our study showed significant variations in the GSH levels across different groups. The higher GSH levels in the group that was given alloxan monohydrate and niacin show that this treatment has a stronger antioxidant effect. In contrast, the reduced GSH levels in rats receiving both *Theobroma cacao *and *Camellia sinensis* warrant further investigation into how these substances interact with cellular antioxidant mechanisms. The present study's results revealed an increase in the GSH levels in diabetic rats after alloxan administration, consistent with previous research studies [16-18].

When ROS is mixed with polyunsaturated fatty acids, they make lipid molecules like 4-hydroxynonenal [19]. This leads to damage to the cell's membrane components, necrosis, and inflammation. The higher MDA levels in the *Theobroma cacao* group indicate worsening oxidative stress, a major cause of diabetes-related complications [20, 21]. Conversely, the reduced MDA levels in the *Camellia sinensis* group suggest its potential as a protective agent against oxidative damage. The present study's results were compared with earlier studies [22, 23].

The body's defense mechanism against free radical-induced damage involves antioxidant enzymes such as SOD and catalase (CAT). SOD catalyzes the conversion of the superoxide anion to hydrogen peroxide and oxygen [24]. The difference in SOD activity, especially the lower activity in the *Theobroma cacao* group, makes us wonder how well some dietary changes help the body's enzymatic antioxidant defense mechanism [25-27]. Our present study's results were corroborated by earlier scientific studies.

The enhanced GSHrd activity in groups receiving *Theobroma cacao* and *Camellia sinensis* suggests that these interventions may improve the cellular antioxidant system's capacity to regenerate GSH, a vital component in combating oxidative stress [28-30].

Decreased blood glucose levels were found in animals treated with metformin compared to other experimentally treated rats, as it induces enzymes participating in glucose oxidation and decreases the utilization of substances used by liver cells to produce glucose. The trends observed in the groups treated with *Theobroma cacao *and *Camellia sinensis*, especially the decreasing glucose levels, open up avenues for further research into the role of these dietary components in glycemic control. Notably, the group receiving both interventions exhibited only a partial response, indicating that the interplay between different dietary substances and diabetes management is intricate and warrants further exploration.

Implications and future research

These findings offer valuable insights into the potential therapeutic effects of *Theobroma cacao* and *Camellia sinensis* diets in diabetic rats. However, the complexity of their effects on oxidative stress markers and blood glucose regulation necessitates further investigation. Mechanistic studies are needed to elucidate the precise pathways through which these dietary components exert their effects.

Additionally, clinical trials in human subjects are warranted to determine the clinical relevance of these findings. Factors such as dosage, duration of treatment, and potential interactions with other therapeutic agents should be carefully considered in future research. A better understanding of the therapeutic potential of these dietary components may open new avenues for complementary strategies to improve oxidative stress management and glycemic control in individuals with diabetes mellitus.

Limitations of the study

The study's limitations include a small sample size and short duration, limiting the generalizability of the findings. Additionally, the research was conducted on rats, which may not fully represent human physiological responses. The specific mechanisms behind the observed effects were not explored, and long-term impacts of *Theobroma cacao* and *Camellia sinensis* diets on diabetes management remain unclear, necessitating further research in diverse populations and over extended periods.

## Conclusions

In our study, we found that the experimental groups that were fed *Theobroma cacao* or *Camellia sinensis* had different effects on oxidative stress parameters and blood glucose levels. Notably, the inducing drug group exhibited elevated glutathione levels, while the combined dietary intervention group showed the lowest. MDA levels varied, with the *Theobroma cacao*-treated diabetic group displaying the highest levels.

These findings underscore the intricate interplay between diet, oxidative stress, and glycemic control in diabetes. The observed variations underscore the need for further research to elucidate the precise mechanisms governing these dietary effects, emphasizing the significance of mechanistic studies. Our study offers valuable insights into the potential of these dietary components to influence oxidative stress and blood glucose regulation in diabetes management.
